# Green space is associated with new-onset stroke among Chinese middle-aged and older adults: data from China Health and Retirement Longitudinal Study (CHARLS)

**DOI:** 10.3389/fpubh.2024.1424510

**Published:** 2025-01-03

**Authors:** Qiong Lu, Cheng Lian, Xinglin Chen

**Affiliations:** ^1^Quyi Research Institute Chinese National Academy of Arts, Beijing, China; ^2^Academic Department, Chinese National Academy of Folk Art, Beijing, China; ^3^Department of Cardiology, Xi’an No.3 Hospital, The Affiliated Hospital of Northwest University, Xi’an, China; ^4^Department of Epidemiology and Biostatistics, Empower U, X&Y Solutions Inc., Boston, MA, United States

**Keywords:** green space, China Health and Retirement Longitudinal Study, aging, stroke, cohort

## Abstract

**Background and aims:**

The relationship between green space and new-onset stroke is inconclusive. This study aimed to investigate the association between green space and stroke risk among Chinese middle-aged and older adults.

**Methods:**

Data were taken from participants aged ≥45 years in the China Health and Retirement Longitudinal Study (CHARLS). Baseline data were collected in 2011 and new-onset stroke data were gathered during follow-up in 2013, 2015, 2018, and 2020. Multivariate Cox regression models were constructed to investigate the association between green space and stroke risk. Subgroup analysis was also performed.

**Results:**

A total of 13,696 participants with a mean age of 59.3 ± 9.3 years were included. After a mean follow-up duration of 6.32 years, there were 728 stroke events during a total of 86,530 person-years of follow-up. The study found a L-shaped relationship between green space and the risk of new-onset stroke in participants. By using a two-piecewise linear regression model, we calculated that the inflection point for the *per capita* park green area was 10.61 square meters per person (log-likelihood ratio test *p* = 0.041). On the left of the inflection point, we observed a negative relationship between green space and the incidence of stroke (HR: 0.89, 95% CI: 0.84–0.94, *p* = 0.0001). On the right side of the inflection point, however, the relationship tended to be saturated (HR: 0.97, 95% CI: 0.94–1.01, *p* = 0.2111).

**Conclusion:**

Our study found that the relationship between green space and the risk of new-onset stroke follows a L-shaped curve. A lower amount of green space is associated with an increased risk of new-onset stroke. These findings require confirmation in future studies.

## Introduction

The rise of industrialization and urbanization worldwide has sparked concerns regarding the correlation between the environment and health. Stroke is a primary cause of disease and mortality, and there is mounting evidence that environmental factors may impact the incidence of stroke (1).

In examining the relationship between green space and the risk of new stroke, an analysis of existing literature reveals a lack of clarity in this area. Although some studies indicate that increased green space may be associated with improved health outcomes, further investigation is required to elucidate the precise mechanisms and extent of this impact. For example, Thompson et al. (2) found that increased green space is associated with reduced stress levels, which may affect health by regulating the hypothalamic–pituitary–adrenal (HPA) axis function, which in turn may affect stroke risk. Furthermore, the study conducted by Wilker et al. (3) indicated that residing in areas with an increased availability of green space was associated with a reduced risk of mortality following a stroke. This finding provides additional evidence in support of the potential health benefits associated with green space (3).

However, the existing literature is inconclusive regarding the precise relationship between green space and the incidence of new strokes. Hsieh et al. (4) highlight the potential for different types of green space to exert varying effects on health, particularly in children and adolescents. Furthermore, they suggest that a lack of green space may be associated with an increased risk of diseases such as asthma. Concurrently, Gascón et al. (5) demonstrated protective associations between green spaces and a range of health outcomes, including cardiovascular disease. However, their systematic review did not explicitly address the impact of green spaces on stroke. This complexity suggests that both the quantity and quality of green space may play a role in different health outcomes, particularly in urban settings.

The relationship between green spaces and health is a topic of great concern in current research. Studies have shown that green spaces are closely linked to mental health, cardiovascular health, and the rehabilitation of mental disorders (6). For instance, research conducted in England and Scotland has found a positive correlation between green spaces and health (6). Li et al. (7) proposed a socio-ecological framework to examine the impact of green spaces on health. According to their research, green spaces can help manage weight, lower blood pressure, and reduce the risk of diseases such as heart disease and diabetes (7). Studies have shown that increasing urban green spaces can significantly improve the health of middle-aged and older adult individuals (8). Moreover, long-term exposure to green spaces has been found to be beneficial to cognitive function. Although the evidence is inconsistent, it still suggests that green space exposure has a positive effect on cognitive function (9, 10). Additionally, the relationship between green space and mental health varies across the life cycle, indicating that green space may be more beneficial to health at certain stages of life (10, 11). Green spaces have been found to be beneficial not only for mental health but also for reducing morbidity and mortality from specific diseases. Studies have shown protective associations between green spaces and diseases such as hypertension, obesity, and coronary heart disease (12). These findings suggest that green spaces have an important role to play in improving health and promoting rehabilitation. The studies provide important references for future urban planning and public policy (6, 12–14).

The relationship between green space and health outcomes, particularly concerning the incidence of new-onset stroke, has garnered significant attention in recent years. Research indicates that exposure to green environments can lead to various health benefits, including improved mental health, increased physical activity, and reduced mortality rates. For instance, a nationwide prospective cohort study found that greater exposure to greenness was associated with lower mortality, primarily through mechanisms such as enhanced mental health and increased social engagement, alongside reduced air pollution exposure and higher levels of physical activity (15). This suggests that green spaces play a crucial role in promoting overall health and well-being, which may, in turn, influence the risk of conditions like stroke.

Moreover, the specific characteristics of green spaces, including their accessibility and quality, are essential in determining their health impacts. Studies have shown that individuals living in areas with ample green space tend to engage more in physical activities, which is a critical factor in reducing cardiovascular diseases and stroke risk (16). For example, research conducted in urban settings has demonstrated a positive correlation between neighborhood greenness and physical activity levels, indicating that access to green spaces encourages healthier lifestyles (17). Additionally, the mental health benefits associated with green spaces, such as stress reduction and improved mood, further underscore their importance in mitigating health risks, including those related to stroke (18).

Despite the established benefits of green spaces, the nuances of their impact on specific health outcomes, such as stroke, remain complex and sometimes contradictory. Some studies have reported no significant associations between proximity to green spaces and the prevalence of cardiovascular conditions, suggesting that other factors, such as socioeconomic status and lifestyle choices, may also play critical roles (19). Furthermore, the interplay between green and blue spaces—such as parks and water bodies—has been less explored, yet emerging evidence indicates that both types of natural environments can contribute positively to mental health and recovery from psychotic disorders (20). This highlights the need for a more comprehensive understanding of how different types of natural environments interact to influence health outcomes.

Although there is evidence of the positive health effects of green spaces, research on the direct relationship between green spaces and new strokes is limited. A recent review of 1,342 papers retrieved as of August 1, 2023, conducted in Medline and Scopus, found only 27 studies that investigated the association between green space and stroke incidence and outcomes using various study designs (cohort, cross-sectional, quasi-experimental, time-stratified case crossover, and ecology). Evidence suggests a protective association between green spaces and stroke-related deaths, with mortality risk ratios ranging from 0.66 to 0.95. Additionally, most studies indicate a negative association between green space and stroke risk, with risk estimates for protective effects ranging from 0.4 to 0.98. However, some studies did not reach statistical significance (21).

There are few studies that directly examine the relationship between green spaces and new strokes. However, a possible link can be inferred from research advances in related fields. It can be speculated that green spaces may indirectly affect the risk of new stroke through improving cardiovascular health, reducing the prevalence of hypertension, and increasing the level of social participation (3). Further studies are required to investigate the direct correlation between green spaces and new strokes, in order to provide stronger evidence to support public health policy-making.

The theoretical framework linking green space to stroke risk involves several interrelated mechanisms that merit further exploration. First, exposure to green spaces has been associated with reduced stress levels, which is a significant risk factor for stroke. Research indicates that living near green environments can lead to lower cortisol levels, thereby mitigating stress-related health issues (22). For instance, a study by Roe et al. demonstrated that increased neighborhood green space was an independent predictor of healthier cortisol patterns, suggesting that green spaces may help regulate neuroendocrine functions that are crucial for maintaining cardiovascular health (23). This regulation of stress hormones can potentially lower blood pressure and improve overall cardiovascular function, thereby reducing the risk of stroke. Additionally, green spaces promote physical activity, which is another critical factor in stroke prevention. Regular physical activity is known to lower the risk of various cardiovascular diseases, including stroke, by improving heart health and reducing obesity (1). Studies have shown that individuals residing in greener neighborhoods are more likely to engage in physical activities, such as walking or cycling, due to the appealing nature of these environments (3). Furthermore, the presence of green spaces can encourage social interactions and community engagement, which have been linked to improved mental health and reduced feelings of isolation—factors that can also contribute to better cardiovascular health (18). Thus, the interplay between green space, stress reduction, and increased physical activity forms a compelling theoretical basis for understanding how green environments may influence stroke risk.

In summary, the current research on the relationship between green spaces and stroke risk reduction is controversial. While some studies suggest a potential protective effect, further research is needed to verify this correlation (21). Currently, the extent of this effect cannot be accurately quantified, and there is a lack of studies on dose–response relationships. For instance, this text explores how to quantify the health benefits of green space and how these benefits vary across different populations (10, 24). Additionally, there are limitations in study design and methodology. Many existing studies use cross-sectional designs, which limit the ability to understand the relationship between green space and health over time (10, 11). Long-term follow-up studies are necessary to confirm these findings.

To date, no studies have focused on the association between green space and stroke risk in Chinese middle-aged and older adults. Based on the above uncertainty, we conducted a prospective cohort study in 450 Chinese communities based on the China Health and Retirement Longitudinal Study (CHARLS) to examine the association between green space and stroke risk.

## Methods

### Study design and population

Data for this study were collected from five periods of the CHARLS in 2011, 2013, 2015, 2018 and 2020. CHARLS is a nationwide questionnaire survey conducted in 150 counties among 28 provinces (autonomous regions and municipalities) in China (25), established by the National Development Research Institute of Peking University. Briefly, participants were included in the baseline survey between June 2011 and March 2012 of middle-aged and older adults who were randomly selected using a probability proportional to size sampling strategy. The baseline questionnaires and physical examinations were carried out on 17,705 participants, and 11,847 participants (67%) completed the blood test. The CHARLS has been approved by the Ethics Committee of Peking University Health Science Center. The Biomedical Ethics Review Board of Peking University approved the CHARLS study (IRB00001052-11015), and all participants provided written informed consent (25). Data from this study and related information can be downloaded from the CHARLS project website.^1^

**Figure 1:**
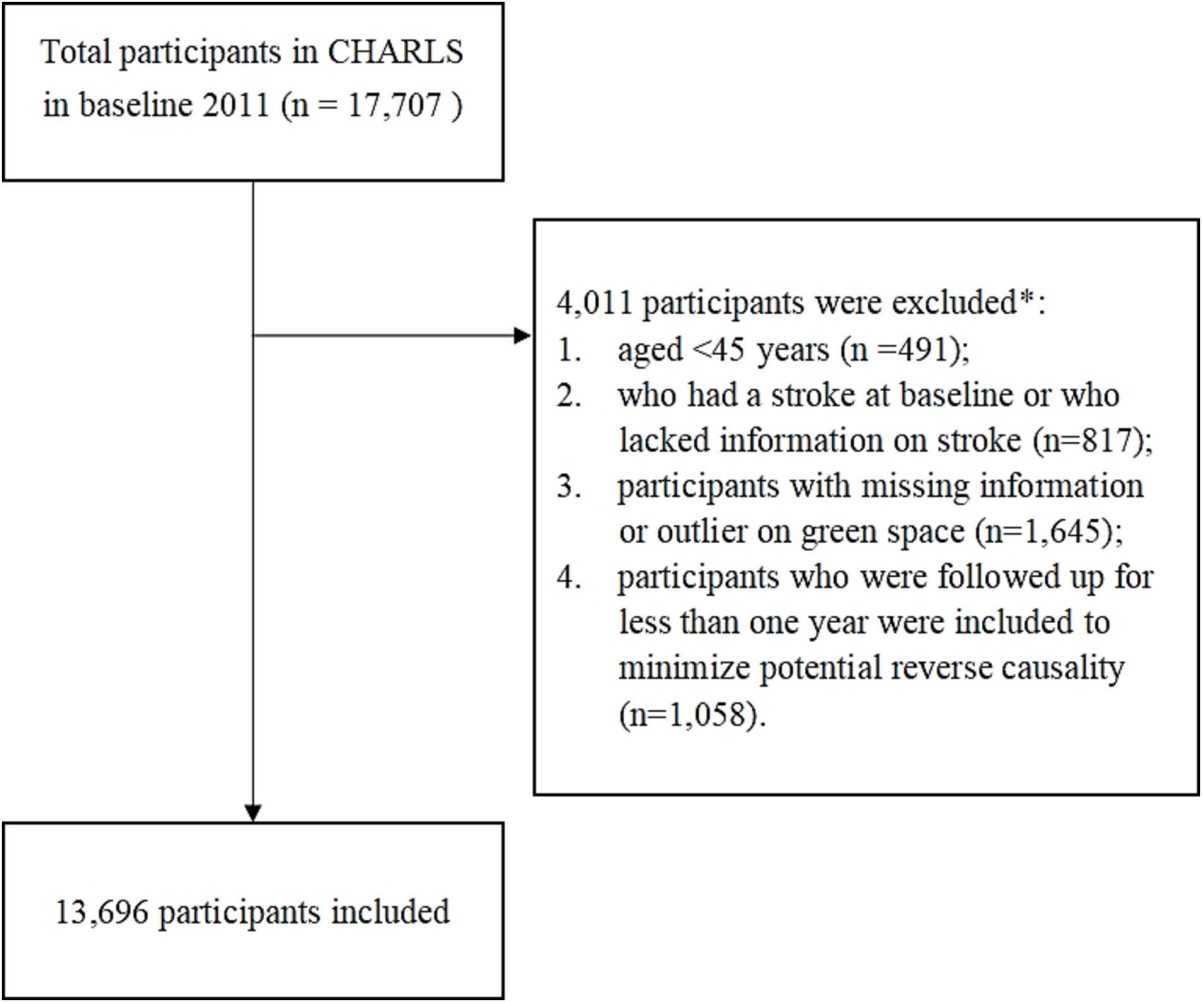
Flowchart of participant selection. *Subjects selected after each step. First, individuals younger than 45 years at wave 1 were excluded, accounting for 491 participants, to focus on an age group more susceptible to the health issues under investigation. Second, we excluded 817 participants who had a stroke at baseline or had missing stroke information, ensuring the study concentrated on new stroke incidences. Third, 1,645 participants with missing or outlier data on green space exposure were excluded to maintain data accuracy. Lastly, to minimize potential reverse causality, we excluded 1,058 participants who were followed for less than 1 year. After applying these criteria, a total of 13,696 individuals were included in the final cohort, as depicted in figure.

For the present study, the following exclusion criteria were applied: (1) aged <45 years old at wave 1 (*n* = 491), (2) had a stroke at baseline or missing information on stroke (*n* = 817), (3) participants with missing information or outlier on green space (*n* = 1,645), (4) participants who were followed up for less than 1 year were included to minimize potential reverse causality (*n* = 1,058). A total of 13,696 individuals meeting these criteria were enrolled in the final cohort (Figure 1). The current study follows the Strengthening the Reporting of Observational Studies in Epidemiology (STROBE) reporting guidelines for cohort studies.

### Study variables

#### Assessment of green space

The CHARLS data set records the address information of the surveyed subjects, including the province, city, and year indicators corresponding to the time of the survey. Furthermore, the data set includes indicators for green space at the provincial, urban area, and temporal levels. It is recommended that the data be consolidated by location and year. The green space area is the independent variable in this study. The representative variable is *per capita* park green space area, which is defined as the total park green area/total population. In order to extract and combine data, another CHARLS-based study was employed (8). In particular, the communityID in the CHARLS database is equivalent to the literal variable of the city code, and the Chinese Statistical Yearbook of Urban Construction also contains the literal variable of the city code, which is merged according to the city code. The data of urban green space from 2011 in the yearbook for analysis and according to the city code and year, the Statistical Yearbook of Urban Construction (Data source: https://www.mohurd.gov.cn/gongkai/fdzdgknr/sjfb/tjxx/index.html).

#### Assessment of stroke

The outcome of the study was a stroke event, defined as a new-onset stroke that occurred during follow-up, using a self-report model. As previously reported (26–28), trained staff asked participants the following standardized questions: (i) Have you been told by your doctor that you have been diagnosed with a stroke? (ii) When was the condition first diagnosed/known to you? (iii) Are you currently receiving any follow-up treatment for your stroke? If the individual answered yes at follow-up, the respondent was classified as having a stroke diagnosis and the self-reported time (year or age) was recorded. To account for the fact that the same person may describe their own time of stroke onset differently in different surveys, the earliest time of stroke onset is used. If no event occurred, the last follow-up time was recorded. Special conditions were used to improve the accuracy of time estimation, as the exact time was not available for all participants. These were taken as the time of the interview - the time of the baseline examination.

### Other covariates

Based on the well-designed questionnaire, the CHARLS-trained interviewers collected information on demographic background, health status, and biomarkers. Covariates were selected on the basis of findings from previous studies and clinical expertise (6, 8, 29, 30). The variables included in this study were age, sex, smoking status (never, current and ever), drinking status (never, current and ever), educational attainment (primary and below primary school, middle school, and high school and above), area of residence (urban and rural), and marital status (married and other), which were collected from each participant in a face-to-face interview as previously described (25). Standing height was measured by a standardized stadiometer (Seca™213, Seca Co., Ltd., Hangzhou, China) and the weight was assessed by a validated scale (Omron™ HN-286 scale, Krill Technology Co., Ltd., Yangzhou, China). BMI was calculated as weight (kg) divided by the square of height (m^2^). Systolic blood pressure (SBP) and diastolic blood pressure (DBP) were recorded three times at least 45 s apart using a digital sphygmomanometer (Omron™ HEM-7200, Dalian, China). Chronic diseases included hypertension, diabetes mellitus (DM) or high blood sugar. Hypertension was defined as SBP ≥140 mmHg and/or DBP ≥90 mmHg or being on antihypertensive therapy. DM was defined as FPG ≥7.0 mmol/L or a history of physician-diagnosed DM.

Age and sex are well-established demographic factors that influence health outcomes, including stroke risk. Studies have consistently shown that older age is associated with a higher incidence of stroke, while sex differences in stroke prevalence and outcomes have been documented, with men generally exhibiting higher rates of stroke at younger ages compared to women (22, 31). Furthermore, lifestyle factors such as smoking and drinking status have been linked to cardiovascular health, with evidence indicating that both current and past smoking significantly increase the risk of stroke (2).

Educational attainment and area of residence are also critical covariates, as they reflect socioeconomic status, which is a known determinant of health disparities. Research indicates that lower educational levels are associated with higher stroke incidence, potentially due to differences in health literacy and access to healthcare resources (7). Additionally, living in urban versus rural areas can influence exposure to environmental stressors and access to healthcare, further impacting stroke risk (1, 5). Marital status has been shown to correlate with health outcomes, where married individuals often report better health and lower mortality rates compared to their unmarried counterparts, likely due to social support mechanisms (3, 16). Collectively, these covariates were selected based on empirical evidence from previous studies, ensuring a comprehensive approach to understanding the multifactorial nature of stroke risk.

### Statistical analysis

Statistical analyses were conducted using the EmpowerStats (www.empowerstats.com, X&Y solutions, Inc. Boston MA) and R software version 3.6.1.^2^ The two-sided alpha level was set at 0.05. Continuous variables were presented using either the mean ± standard deviation (SD) (for normally distributed data) or median interquartile ranges (IQR) (for skewed distribution). Categorical data are presented as counts with percentages.

Based on the tertile of green space, participants were divided into three groups (T1, ≤9.71; T2, 9.71–≤12.50; T3, >12.50), the differences between green space groups was compared using one-way analysis of variance (ANOVA) (for normally distributed data), the Kruskal–Wallis H test (for skewed data) for continuous data and chi-squared tests for categorical variables. We further visualized our findings by generating Kaplan–Meier plots based on green space groupings.

Univariate and multivariate Cox proportional-hazards regression model analyses were used. The results are presented as hazard ratio (HR) with its 95% confidence intervals (95% CIs). We selected these confounders on the basis of their association with the outcomes of interest or changes in effect estimates of more than 10% (32). After considering the clinical significance, we adjusted for the following covariates: age (years), sex, area of residence, education, marital status, body mass index, smoking, drinking, hypertension, and number of parks.

To explore potential non-linear associations between green space and risk of stroke, we used a generalized additive model (GAM) with smooth curve fitting. We then used a two-piece-wise linear regression model to examine the threshold effect of green space on risk of stroke. We also performed a log-likelihood ratio test and compared the one-line linear regression model with the two-piece-wise linear model as described in the previous analysis (33, 34). We used the bootstrap resampling method to calculate the 95% CI for the turning point (35).

Sensitivity analysis was utilized to test the robustness of our findings. (1) Previous studies have shown a significant association between hypertension and stroke (36). Participants without hypertension (n = 10,615) were analyzed. (2) Dummy variables were used to indicate missing covariate values, which was performed when continuous variables were missing more than 2% of value, as described in the previous analysis (37, 38). (3) The E-value was calculated to assess the possibility of unmeasured confounders between green space and stroke risk (39). The E-value quantifies the amount of unmeasured confounding that is required to eliminate the observed association between green space and stroke risk. (4) To examine the robustness of our results, subgroup analyses of different subgroups (age, body mass index, systolic blood pressure, diastolic blood pressure, number of parks, sex, area of residence, education, marital status, smoking, drinking, hypertension, dyslipidemia, and diabetes or hyperglycaemia) were performed using stratified Cox proportional hazards regression models. To assess the presence of an interaction term, we used likelihood ratio tests in models with and without an interaction term.

## Results

### Characteristics of the participants

A total of 13,696 individuals with a mean age of 59.3 ± 9.3 years were included in the study. The cohort was categorized into three groups (T1, T2, and T3) based on the tertile of green space. The baseline characteristics are summarized in Table 1. Almost 78.7% of all participants lived in rural areas, and more than 66.6% of the respondents were primary illiterate or less. Compared to the subjects in the group with the lowest amount of green space, those in the higher groups had a greater number of parks. Significant differences were observed among the three groups in terms of height, BMI, systolic blood pressure, diastolic blood pressure, area of residence, education level, and previous history of hyperlipidemia (all *p* < 0.05).

**Table 1:** Baseline characteristics and new-onset stroke according to green space (China Health and Retirement Longitudinal Study 2011–2020).

Variable	All participants	*Per capita* park green area tertile (square meters/person)			
		≤9.71	9.71 to ≤12.50	>12.50	*p*-value
N	13,696	4,397	4,653	4,646	
Age (years)	59.3 ± 9.6	59.2 ± 9.5	59.5 ± 9.8	59.2 ± 9.5	0.374
Height (cm)	158.0 ± 8.6	157.7 ± 8.7	158.2 ± 8.6	158.2 ± 8.5	0.024
Weight (kg)	58.6 ± 11.7	58.6 ± 11.5	58.3 ± 11.3	58.9 ± 12.2	0.136
Body mass index (kg/m^2^)	23.4 ± 3.9	23.5 ± 3.8	23.3 ± 3.8	23.5 ± 4.1	0.029
SBP (mmHg)	132.3 ± 22.5	131.0 ± 22.2	133.3 ± 22.4	132.6 ± 22.8	<0.001
DBP (mmHg)	76.4 ± 12.7	75.9 ± 12.7	76.6 ± 12.3	76.7 ± 13.1	0.022
Number of parks	13.0 (8.0–27.0)	11.0 (7.0–15.0)	16.0 (9.0–33.0)	17.0 (11.0–32.0)	<0.001
*Per capita* park green area square meters/person	11.5 ± 3.1	8.3 ± 1.3	11.0 ± 0.8	15.1 ± 1.6	<0.001
Sex (%)					0.521
Male	6,643 (48.5)	2,136 (48.6)	2,229 (47.9)	2,278 (49.1)	
Female	7,042 (51.5)	2,259 (51.4)	2,422 (52.1)	2,361 (50.9)	
Area of residence (%)					<0.001
Rural Hukou	10,784 (78.7)	3,432 (78.1)	3,706 (79.6)	3,646 (78.5)	
Urban Hukou	2,809 (20.5)	935 (21.3)	929 (20.0)	945 (20.3)	
Missing	103 (0.8)	30 (0.7)	18 (0.4)	55 (1.2)	
Education (%)					0.004
Primary and below	9,112 (66.6)	2,872 (65.3)	3,089 (66.5)	3,151 (67.9)	
Middle school	2,865 (20.9)	968 (22.0)	1,007 (21.7)	890 (19.2)	
High school and above	1709 (12.5)	556 (12.6)	551 (11.9)	602 (13.0)	
Marital status (%)					0.407
Married	12,028 (87.8)	3,882 (88.3)	4,087 (87.8)	4,059 (87.4)	
Other	1,668 (12.2)	515 (11.7)	566 (12.2)	587 (12.6)	
Smoking (%)					0.279
Never	8,233 (60.2)	2,671 (60.8)	2,830 (60.9)	2,732 (58.9)	
Current	4,372 (32.0)	1,383 (31.5)	1,457 (31.4)	1,532 (33.0)	
Ever	1,070 (7.8)	336 (7.7)	359 (7.7)	375 (8.1)	
Drinking (%)					0.480
Current	3,534 (25.9)	1,166 (26.6)	1,175 (25.3)	1,193 (25.7)	
Ever	1,105 (8.1)	343 (7.8)	368 (7.9)	394 (8.5)	
Never	9,029 (66.1)	2,880 (65.6)	3,100 (66.8)	3,049 (65.8)	
Hypertension (%)					0.081
No	10,615 (78.0)	3,417 (78.1)	3,656 (78.9)	3,542 (76.9)	
Yes	3,000 (22.0)	960 (21.9)	979 (21.1)	1,061 (23.1)	
Dyslipidemia (%)					0.021
No	12,278 (91.5)	3,963 (91.2)	4,130 (90.8)	4,185 (92.4)	
Yes	1,142 (8.5)	382 (8.8)	416 (9.2)	344 (7.6)	
Diabetes or hyperglycaemia (%)					0.605
No	12,845 (94.8)	4,126 (94.6)	4,380 (95.1)	4,339 (94.7)	
Yes	703 (5.2)	234 (5.4)	227 (4.9)	242 (5.3)	
New-onset stroke (%)					<0.001
No	12,968 (94.7)	4,106 (93.4)	4,433 (95.3)	4,429 (95.3)	
Yes	728 (5.3)	291 (6.6)	220 (4.7)	217 (4.7)	

Data are expressed as mean ± SD, median (25th-75th percentile) or percentage. Among the 13,696 participants, the number of missing values for the covariates was 2,737 (20.0%) for Height, 2,699 (19.7%) for weight, 2,794 (20.4%) for body mass index, 2,677 (19.5%) for systolic blood pressures, 2,678 (19.6%) for diastolic blood pressure, 11 (0.1%) for sex, 103 (0.8%) for area of residence, 10 (0.1%) for education, 21 (0.2%) for smoking, 28 (0.2%) for drinking, 81 (0.6%) for hypertension, 276 (2.0%) for dyslipidaemia and 148 (1.1%) for diabetes or hyperglycaemia. SBP Systolic blood pressure; DBP Diastolic blood pressure.

### The incidence rate of stroke

After a mean follow-up duration of 6.32 years, there were 728 stroke events during a total of 86,530 person-years of follow-up. This corresponds to a prevalence of new-onset stroke of 5.32% (*N* = 728). Among the three green space groups (≤9.71 square meters/person, 9.71 to ≤12.50 square meters/person, and > 12.50 square meters/person), the number of new-onset stroke observed was 291 (6.62%), 220 (4.73%), and 217 (4.67%), respectively (Table 1).

### Factors influencing the risk of stroke analyzed by univariate cox proportional hazards regression

Based on univariate analyses, the risk of stroke was correlated with age, education, marital status, smoking status, weight, body mass index, systolic blood pressure, diastolic blood pressure, hypertension, dyslipidemia, diabetes or hyperglycaemia, and number of parks (all *p* < 0.05) (Table 2).

**Table 2:** The unadjusted association between baseline variables and risk of stroke (total *N* = 13,573).

Exposure	Statistics	HR (95% CI)	*P* value
Age (years)	59.3 ± 9.6	1.03 (1.03, 1.04)	<0.0001
Sex (%)			
Male	6,643 (48.54%)	Reference	
Female	7,042 (51.46%)	0.88 (0.76, 1.01)	0.0749
Area of residence			
Rural	10,784 (78.74%)	Reference	
Urban	2,809 (20.51%)	1.15 (0.96, 1.37)	0.1194
Missing	103 (0.75%)	1.78 (0.92, 3.44)	0.0861
Education			
Primary and below	9,112 (66.58%)	Reference	
Middle school	2,865 (20.93%)	0.75 (0.61, 0.91)	0.0035
High school and above	1709 (12.49%)	0.81 (0.64, 1.03)	0.0895
Marital status recoded			
Married	12,028 (87.82%)	Reference	
Other	1,668 (12.18%)	1.54 (1.27, 1.88)	<0.0001
Smoking			
Never	8,233 (60.20%)	Reference	
Current	4,372 (31.97%)	1.09 (0.93, 1.28)	0.3010
Ever	1,070 (7.82%)	1.62 (1.28, 2.05)	<0.0001
Drinking			
Current	3,534 (25.86%)	Reference	
Ever	1,105 (8.08%)	0.80 (0.59, 1.09)	0.1540
Never	9,029 (66.06%)	0.90 (0.77, 1.07)	0.2339
Height (cm)	158.02 ± 8.61	1.00 (0.99, 1.01)	0.4844
Weight (kg)	58.60 ± 11.67	1.01 (1.01, 1.02)	0.0003
Body mass index (kg/m2) tertile group			
12.2–21.5	3,633 (33.32%)	Reference	
21.5–24.5	3,625 (33.25%)	1.13 (0.91, 1.40)	0.2583
24.5–62.8	3,644 (33.43%)	1.57 (1.29, 1.92)	<0.0001
Systolic blood pressure (mmHg) group			
< 140	7,454 (67.65%)	Reference	
≥ 140	3,565 (32.35%)	1.97 (1.67, 2.31)	<0.0001
Diastolic blood pressure (mmHg) group			
< 90	9,433 (85.61%)	Reference	
≥ 90	1,585 (14.39%)	1.79 (1.48, 2.17)	<0.0001
Hypertension			
No	10,615 (77.97%)	Reference	
Yes	3,000 (22.03%)	2.45 (2.11, 2.84)	<0.0001
Dyslipidemia			
No	12,278 (91.49%)	Reference	
Yes	1,142 (8.51%)	1.84 (1.49, 2.27)	<0.0001
Diabetes or hyperglycaemia			
No	12,845 (94.81%)	Reference	
Yes	703 (5.19%)	1.69 (1.30, 2.21)	0.0001
Number of park tertile group			
2–8	3,628 (26.49%)	Reference	
9–19	5,303 (38.72%)	0.83 (0.70, 0.98)	0.0325
20–789	4,765 (34.79%)	0.67 (0.56, 0.81)	<0.0001

Data are expressed as the mean ± SD, or counts with percentages. HR, Hazard ratios, CI, confidence.

### Relationship between green space and the risk of stroke

**Figure 2:**
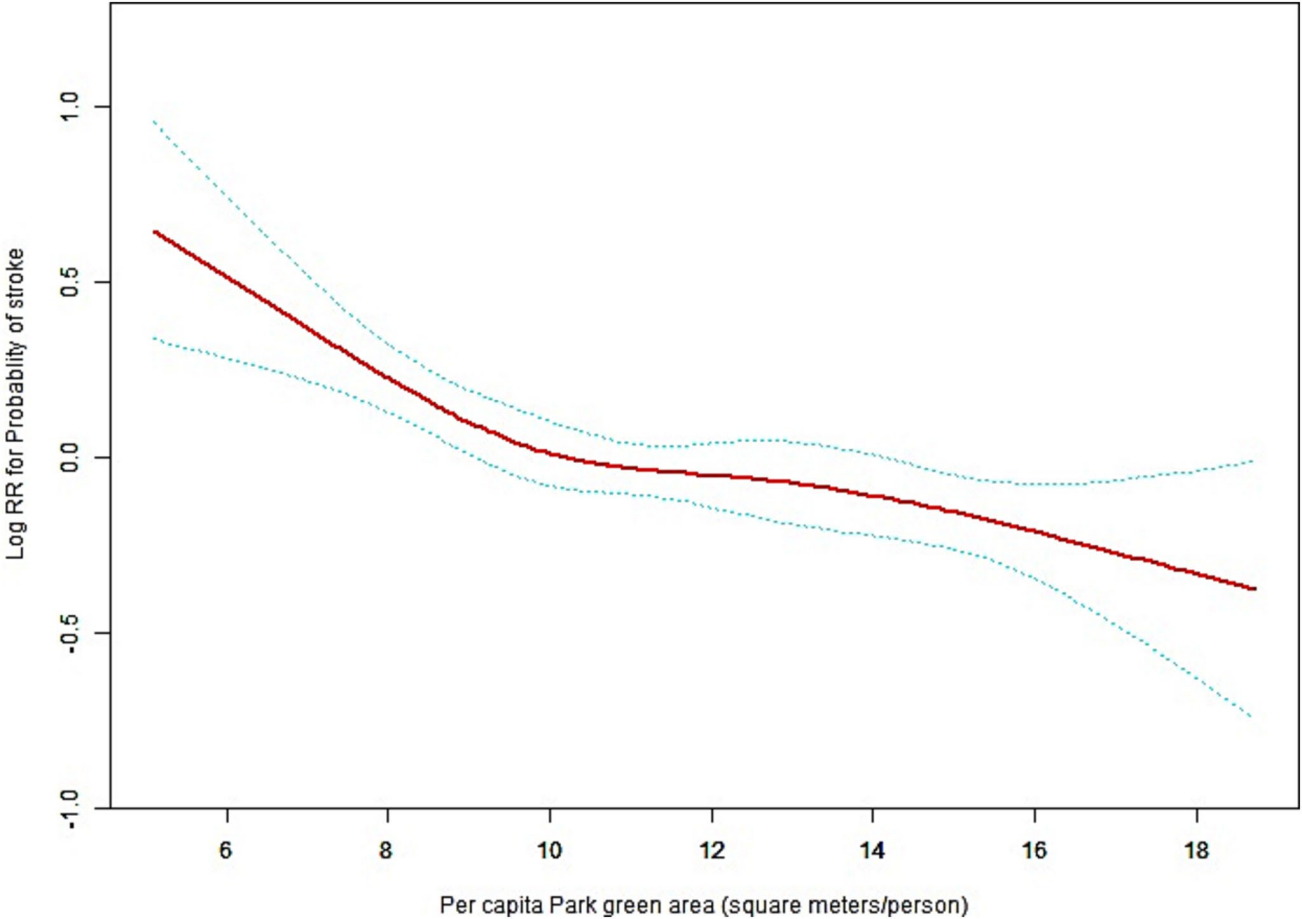
General additive models (GAM) demonstrate the relationship between green space and the risk of stroke. The resulting figures show the log(relative risk) in the y-axis and the continuous covariate in the x-axis. Solid rad line represents the smooth curve fit between variables. Blue bands represent the 95% of confidence interval from the fit. Adjusted for age (years)(Smooth), sex, area of residence, education, marital status, body mass index (Smooth), smoking, drinking, hypertension, and number of parks. Restricted cubic spline smoothing were applied.

The generalized additive model showed a L–shaped association between green space and risk of stroke (Figure 2). This L-shaped association suggests that there is a significant reduction in stroke risk with an initial increase in green space exposure, indicating a steep decline in risk at lower levels of green space. However, as the amount of green space continues to increase, the rate of risk reduction diminishes, eventually plateauing. This pattern implies that while increasing green space is beneficial for reducing stroke risk, the most substantial benefits are observed with initial increases, and additional green space beyond a certain point yields diminishing returns in terms of further risk reduction. This nuanced understanding of the relationship highlights the importance of ensuring adequate green space in urban planning to maximize public health benefits. By using a two-piecewise linear regression model, we calculated that the inflection point for the *per capita* park green area was 10.61 square meters per person (log-likelihood ratio test *p* = 0.041). On the left of the inflection point, we observed a negative relationship between green space and the incidence of stroke (HR: 0.89, 95% CI: 0.84–0.94, *p* = 0.0001). On the right side of the inflection point, however, the relationship tended to be saturated (HR: 0.97, 95% CI: 0.94–1.01, *p* = 0.2111) (Table 3).

**Table 3:** Threshold effect analysis of green space and risk of new-onset stroke (total *N* = 13,573).

Models	*Per capita* park green area (square meters/person)	
	HR (95%CI)	*P* value
Model I		
One line effect	0.94 (0.92, 0.97)	<0.0001
Model II		
Turning point (K)	10.61	
Green space < K	0.89 (0.84, 0.94)	0.0001
Green space ≥ K	0.97 (0.94, 1.01)	0.2111
*P* value for LRT test*		0.041
95% CI for turning point	10.2–11.12	

Data were presented as HR (95% CI) p value; Model I, linear analysis; Model II, non-linear analysis. CI, confidence interval; HR, hazard ratio; LRT, logarithm likelihood ratio test. Adjusted for age (years) (Smooth), sex, area of residence, education, marital status, body mass index (Smooth), smoking, drinking, hypertension, and number of parks. *P < 0.05 indicates that model II is significantly different from Model I. Restricted cubic spline smoothing were applied. For 13,696 participants, covariates with missing values > 2% were treated with dummy variables. These included body mass index. Covariates with missing values < 2%, such as sex, area of residence, education, smoking, drinking, and hypertension, led to the exclusion of 123 participants. The final sample size for the model was 13,573 participants.

The linear regression model and a two-piece-wise linear regression model were compared, and the *p* value of the log-likelihood ratio test was 0.041. The 95% CI for turning point of green space was 10.2–11.12 square meters per person (Table 3). This result indicates that the two-piece-wise linear regression model should be used to fit the model.

**Figure 3:**
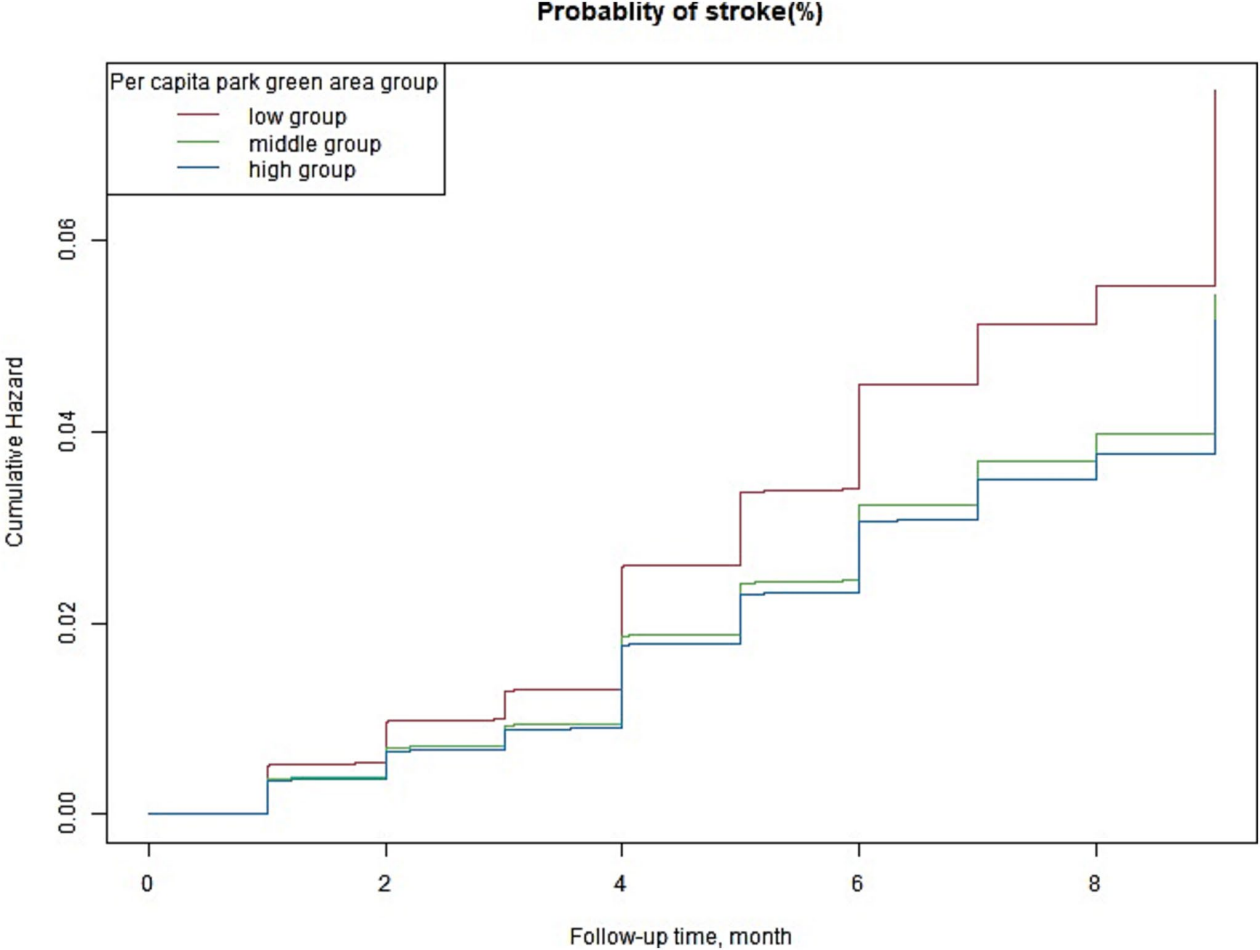
Multivariable-adjusted Kaplan–Meier plot for the association of the green space groups with the incidence of stroke; Kaplan–Meier plot adjusted for age (years), sex, area of residence, education, marital status, body mass index, smoking, drinking, hypertension, and number of parks.

Kaplan–Meier curves showed that patients within the lower green space categories (the high group) had a higher cumulative incidence of stroke (Figure 3). The curves distinctly illustrated that patients residing in areas categorized as having lower levels of green space, referred to as the “high group” in terms of stroke incidence, experienced a noticeably increased cumulative incidence of stroke over the study period. This finding suggests a significant correlation between reduced access to green spaces and higher stroke risk, with those in less green environments seeing a steeper and faster accumulation of stroke cases compared to individuals in more verdant settings.

### Sensitive analysis

The sensitivity analysis was consistent with that of the main analysis. Firstly, in a group without hypertension, consisting of 10,615 participants, the analysis adjusted for factors such as age, sex, area of residence, education, marital status, body mass index, smoking, drinking, and number of parks. This analysis still showed consistent results with that of the main analysis (Supplementary Figure S1 and Table 1). Secondly, Dummy variables were used to indicate missing covariate values. Similar results were obtained after considering the impact of missing data (data not shown).

Furthermore, we generated an E-value to assess the sensitivity to unmeasured confounding. The primary findings were robust, unless there was an unmeasured confounder with an HR greater than 1.74. Based on all sensitivity analyses, our findings were robust.

The association between green space and stroke risk was not influenced by age, body mass index, systolic blood pressure, diastolic blood pressure, number of parks, sex, area of residence, education, marital status, smoking, alcohol consumption, hypertension, dyslipidaemia, and diabetes or hyperglycaemia in any of the prespecified or exploratory subgroups examined (Table 4). That is, the interaction between these variables and green space was not statistically significant (*p* > 0.05 for interaction).

**Table 4:** Stratified associations between green space and stroke.

Characteristics	No of participants	HR (95%CI)	*P* value	*P* for interaction
Age (years) categorical				0.8634
< 60	7,936	0.94 (0.90, 0.97)	0.0009	
≥ 60, <70	3,725	0.94 (0.90, 0.98)	0.0039	
≥ 70, <80	1,631	0.95 (0.90, 1.01)	0.1071	
≥ 80	404	0.90 (0.80, 1.02)	0.0957	
Sex (%)				0.7706
Male	6,643	0.94 (0.91, 0.97)	0.0002	
Female	7,042	0.95 (0.91, 0.98)	0.0023	
Area of residence				0.1888
Rural	10,784	0.93 (0.90, 0.96)	<0.0001	
Urban	2,809	0.98 (0.93, 1.04)	0.5428	
Missing	103	0.97 (0.70, 1.36)	0.8742	
Education				0.9891
Primary and below	9,112	0.94 (0.91, 0.97)	<0.0001	
Middle school	2,865	0.95 (0.89, 1.01)	0.1003	
High school and above	1709	0.94 (0.87, 1.02)	0.1347	
Marital status				0.7489
Married	12,028	0.95 (0.92, 0.97)	0.0001	
Other	1,668	0.92 (0.86, 0.97)	0.0037	
Smoking				0.6176
Never	8,233	0.93 (0.90, 0.96)	<0.0001	
Current	4,372	0.95 (0.91, 1.00)	0.0289	
Ever	1,070	0.96 (0.89, 1.03)	0.2535	
Drinking				0.9887
Current	3,534	0.94 (0.90, 0.99)	0.0148	
Ever	1,105	0.96 (0.88, 1.05)	0.4030	
Never	9,029	0.94 (0.91, 0.97)	<0.0001	
Body mass index (kg/m^2^) tertile group				0.8515
12.2–21.5	3,633	0.94 (0.89, 0.98)	0.0102	
21.5–24.5	3,625	0.92 (0.88, 0.97)	0.0022	
24.5–62.8	3,644	0.95 (0.91, 0.99)	0.0242	
Systolic blood pressure (mmHg) group				0.3773
< 140	7,454	0.93 (0.89, 0.97)	0.0002	
≥ 140	3,565	0.95 (0.91, 0.99)	0.0175	
Diastolic blood pressure (mmHg) group				0.9331
< 90	9,433	0.94 (0.91, 0.97)	0.0001	
≥ 90	1,585	0.94 (0.89, 1.00)	0.0413	
Hypertension				0.1770
No	10,615	0.93 (0.90, 0.96)	<0.0001	
Yes	3,000	0.95 (0.92, 0.99)	0.0156	
Dyslipidemia				0.9131
No	12,278	0.94 (0.91, 0.96)	<0.0001	
Yes	1,142	0.94 (0.88, 1.01)	0.0888	
Diabetes or hyperglycaemia				0.9172
No	12,845	0.94 (0.92, 0.97)	<0.0001	
Yes	703	0.93 (0.85, 1.01)	0.0880	
Park number tertile group				0.5573
Low	3,628	0.96 (0.91, 1.02)	0.1751	
Middle	5,303	0.97 (0.93, 1.01)	0.1693	
High	4,765	0.94 (0.90, 0.98)	0.0052	

Above model adjusted for age (years), sex, area of residence, education, marital status, body mass index, smoking, drinking, hypertension, and number of parks. In each case, the model is not adjusted for the stratification variable. HR, Hazard ratios; CI, confidence.

## Discussion

This cohort study found a lower green space was associated with a higher risk of new-onset stroke in a large sample of middle-aged and older Chinese adults. The major finding was that the association between green space and the risk of new-onset stroke was L-shaped, and the risk was highest in those with very low green spaces. To our knowledge, this is the first study to report a nonlinear relationship between green space and risk of new-onset stroke in among Chinese middle-aged and older adults. Our findings need to be confirmed in future studies.

In previous studies, 136 (1.98%) stroke events occurred in the CHARLS cohort of 6,877 Chinese participants aged 45–90 years with a mean follow-up of 4 years (40). The incidence risk of stroke is relatively low, possibly because of the relatively short follow-up (40). In this study, after a mean follow-up of 6.32 years, there were 728 stroke events during a total of 86,530 person-years of follow-up. This corresponds to a prevalence of new-onset stroke of 5.32%. We acknowledge that the participants limit the generalizability of the results to other populations.

The effect of green space on stroke risk has been supported by other studies. For example, a meta-analysis and systematic review published in 2023 by found that there is likely to be some protective effect of green space on stroke, with the benefits most convincingly shown for post-stroke outcomes (21). The meta-analysis showed that green space was inversely associated with stroke risk, with risk estimates from studies showing a protective effect ranging between 0.4 and 0.98; however, results were more mixed and some did not reach statistical significance (21). Our results showed that when green space was <10.61 square meters/person, the risk of stroke rate decreased with an adjusted HR of 0.89 (95% CI: 0.84–0.94, *p* = 0.0001). Our study is consistent with previous research, and the significant association is mainly due to the identification of piecewise relationships, particularly the detection of non-linear relationships.

A recent nationwide cohort study published in 2024 analysed 18,344,976 years of follow-up and identified 94,256 stroke cases caused by multiple pollutants. After adjusting for air pollution and noise, the study found no association between green space at the residence and the risk of stroke (1). To the best of our knowledge, although there has been concern about the relationship between green spaces and the risk of stroke, the association between the two is still unclear. This prompted us to conduct the current study. We observed a nonlinear dose–response relationship between green space and risk of new-onset stroke. Our results showed that when green space was ≥10.61 square meters/person, the HR for risk of stroke was 0.97 (95% CI: 0.94–1.01, *p* = 0.2111). When studying the relationship between green space and stroke, it is recommended to consider the dose–response relationship, which is a nonlinear relationship.

Although the precise mechanisms are not fully understood, the relationship between green space and stroke risk is potentially linked.

The relationship between green space and stroke risk is a complex issue that has garnered significant attention in recent research. While the precise mechanisms underlying this relationship are not yet fully understood, several studies have delved into various aspects of this association. Green spaces play a crucial role in reducing the risk of strokes through various mechanisms. Firstly, they improve air quality, which is essential for cardiovascular health (41). Green spaces also help in relieving psychological stress, promoting physical activity, and providing a rich source of nutrients (42). Studies have shown that living in areas with higher amounts of green spaces reduces mortality, mainly due to cardiovascular diseases (5). Additionally, green spaces have been linked to the restoration from stress and mental fatigue, indicating their positive impact on mental health (43). Exposure to green spaces triggers rapid psychological, physiological, and endocrinological effects compared to urban environments (44). Furthermore, the relationship between distance to green spaces and participation in physical activity has been explored in various studies (45). Green spaces not only encourage physical activity but also contribute to social interaction and improved air quality, all of which are beneficial for cardiovascular health (41). A study of urban squares in Bandung, Indonesia, demonstrated that the square, as a public space, attracts visitors from diverse geographical regions and fosters social interaction (46). The formation of positive social interactions has been demonstrated to reinforce social support networks and enhance mental wellbeing. This, in turn, may serve to mitigate the risk of stroke. While the study is primarily concerned with the efficacy of regulatory enforcement, its context and methodology offer significant insights into the role of green space in public health. A more detailed examination of the role of green space in stress reduction, social interaction and physical activity could enhance our comprehension of the manner in which these processes influence the risk of stroke. This not only enhances the existing body of literature but also provides empirical evidence to inform the development of public health policies, underscoring the significance of green space in urban planning. The Long City Square study provides a framework for evaluating the use of public space through qualitative descriptive methods (46), which can also be applied to studying the health effects of green space. In conclusion, the multifaceted benefits of green spaces in reducing the risk of strokes by enhancing air quality, alleviating stress, promoting physical activity, and supporting overall well-being are well-documented in scientific literature. Our study did not collect data on air quality, psychological stress, or physical activity. It was observed that areas with lower green space had fewer parks. Further investigation is needed to determine if the number of parks is associated with relevant indicators such as air quality, psychological stress, and physical activity, and to explain the increased risk of risk among patients with low green space. The cohort study presented here is based on the CHARLS database and may provide valuable insights into the relationship between green spaces and the health of middle-aged and older adults in China.

### Study limitations

A common problem in observational studies is unmeasured confounders. As seen in Table 1, compared to the subjects in the group with the lowest amount of green space, those in the higher groups had a greater number of parks. Significant differences were observed among the three green space groups in terms of height, BMI, systolic blood pressure, diastolic blood pressure, area of residence, education level, and previous history of hyperlipidemia (all *p* < 0.05). These differences may be indicative of unmeasured confounders, such as income and medical insurance, which may affect the risk of stroke. Although we adjusted for possible confounding factors, including age, sex, area of residence, education, marital status, body mass index, smoking, drinking, hypertension, and number of parks. Additional limitations of our study include missing data for some variables. Nevertheless, we used contemporary methods to deal with missing data to minimize bias. An important area for improvement was the potential confounding factors that could influence the observed association between green space and stroke incidence. Although we controlled for several socio-demographic and health-related variables, there are other environmental and lifestyle factors that could confound the results, such as air pollution levels, access to healthcare, and physical activity patterns. Among the analyzable indicators included in this study, there are currently no direct indicators of air pollution levels, access to health care and physical activity patterns, which is one of the limitations of this study. The results of this study are consistent after stratification by area of residence and education, which may to some extent reflect the influence of different living environments.

Another limitation is related to the fact that the diagnosis of new-onset stroke was based on a self-reported questionnaire, which may introduce the possibility of misclassification. Unfortunately, we did not have information on the specific type of stroke, which prevented us from distinguishing between haemorrhagic and ischaemic strokes. Moreover, the cross-sectional nature of the study precluded the possibility of establishing causality with certainty. We propose the implementation of specific longitudinal analyses or experimental designs that could more definitively elucidate the causal pathways involved.

Furthermore, the lack of information on interventions during the initial stabilization period could have influenced the risk of stroke. It is important to note that the potential effects of these interventions would likely bias towards null, leading to an underestimation of the association between green space and stroke risk.

Finally, it is acknowledged that the study participants were middle-aged and older Chinese adults who were referred to, which may limit the generalizability of the findings to other populations. The study’s focus on a specific demographic group in China means that while the results are significant for this population, their applicability to other cultural or geographical contexts may be limited. This limitation highlights the importance of considering cultural, social, and environmental factors that may influence the outcomes in different populations. To address these limitations, future research should aim to explore similar associations in diverse settings, including different age groups, ethnicities, and regions. Such studies could provide a more comprehensive understanding of the phenomena under investigation and help determine whether the observed patterns hold true across various contexts. Additionally, cross-cultural studies could offer insights into the universal and culture-specific aspects of the findings, thereby enhancing the robustness and applicability of the research. By expanding the scope of research to include a broader range of participants, scholars can contribute to a more nuanced and globally relevant body of knowledge.

## Conclusion

The study analysed data from the CHARLS database and identified 13,696 participants. Our findings reveal a non-linear dose–response relationship between the availability of green spaces and the risk of new-onset stroke, characterized by an L-shaped curve. Specifically, we observed that lower amounts of green space are significantly associated with an increased risk of stroke, suggesting that individuals residing in areas with limited access to green environments may be at greater risk for this age-related health condition. These results underscore the critical importance of considering green space availability as a modifiable environmental factor in public health strategies aimed at reducing stroke incidence, particularly among older adults. Given the growing body of evidence linking green spaces to various health outcomes, our findings advocate for the integration of green space planning and development into urban policy frameworks. By enhancing access to green environments, we may not only improve the overall quality of life for aging populations but also mitigate the risk of stroke and other related health issues. Future research should further explore the mechanisms underlying this relationship and assess the potential benefits of green space interventions in diverse populations.

## Data Availability

Publicly available datasets were analyzed in this study. The datasets presented in this study can be found in online repositories. The names of the repository/repositories and accession number(s) can be found below: http://charls.pku.edu.cn/ and https://www.mohurd.gov.cn/gongkai/fdzdgknr/sjfb/tjxx/index.html.

## Footnotes

^1^ http://charls.pku.edu.cn/

^2^ http://www.r-project.org
