# Supplementary material for: Green space is associated with new-onset stroke among Chinese middle-aged and older adults: data from China Health and Retirement Longitudinal Study (CHARLS)
Source: Front Public Health. 2025 Jan 3;12:1424510. doi: 10.3389/fpubh.2024.1424510 (PMC11738923; doi:10.3389/fpubh.2024.1424510)
Supplement: Supplementary file 1 [file Supplementary_file_1.doc]

**Title page**

**Green space is associated with new-onset stroke among Chinese middle-aged and older adults: data from China health and retirement longitudinal study (CHARLS)**

Cheng Lian^1^, Qiong Lu ^2,3*^, Xinglin Chen^3,4^

^1^Department of Cardiology, Xi’an No.3 Hospital, the Affiliated Hospital of Northwest University, Xi’an, Shaanxi, China; ^2^Quyi Research Institute, Chinese National Academy of Arts, No.81, Laiguangying West Road, Chaoyang District, Beijing, China; ^3^Academic Department, Chinese National Academy of Folk Art, No.81, Laiguangying West Road, Chaoyang District, Beijing, China; ^4^Department of Epidemiology and Biostatistics, Empower U, X&Y Solutions Inc., Boston, MA, United States.

^*^Corresponding author

E-mail: luqiong@cflac.net

Supplementary material

# Supplemental material S1


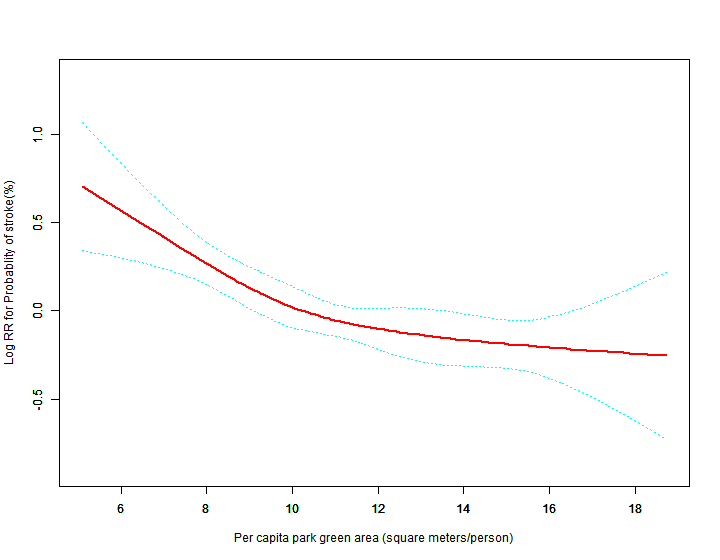


**Figure S1** General additive models (GAM) demonstrate the relationship between green space and the risk of stroke in participants without hypertension (N=10,615). The resulting figures show the log(relative risk) in the y-axis and the continuous covariate in the x-axis. Solid rad line represents the smooth curve fit between variables. Blue bands represent the 95% of confidence interval from the fit. Adjusted for age (years)(Smooth), sex, area of residence, education, marital status, body mass index (Smooth), smoking, drinking, and park number. Restricted cubic spline smoothing were applied.

# Supplemental material S2

**Table S1** Threshold effect analysis of green space and risk of new-onset stroke in participants without hypertension (N=10,615)

| Models | Per capita park green area (square meters/person) | |
| --- | --- | --- |
|  | HR (95%CI) | *P* value |
| Model I |  |  |
| One line effect | 0.93 (0.91, 0.97) | <0.0001 |
| Model II |  |  |
| Turning point (K) | 10.73 |  |
| green space < K | 0.86 (0.80, 0.93) | <0.0001 |
| green space ≥ K | 0.99 (0.93, 1.04) | 0.6013 |
| *P* value for LRT test* |  | 0.019 |
| 95% CI for turning point | 10.3-11.42 | |

Data were presented as HR (95% CI) *P* value; Model I, linear analysis; Model II, non-linear analysis. CI, confidence interval; HR, hazard ratio; LRT, logarithm likelihood ratio test.

Adjusted for age (years)(Smooth), sex, area of residence, education, marital status, body mass index (Smooth), smoking, drinking, and park number.

* *P*<0.05 indicates that model II is significantly different from Model I. Restricted cubic spline smoothing were applied.

For 10,615 participants, covariates with missing values >2% were treated with dummy variables.

These included body mass index. Covariates with missing values <2%, such as sex, area of residence, education, smoking, and drinking, led to the exclusion of 32 participants. The final sample size for the model was 10,583 participants.
